# The Relationship Between Type 2 Diabetes Mellitus and Related Thyroid Diseases

**DOI:** 10.7759/cureus.20697

**Published:** 2021-12-25

**Authors:** Suha Majeed Mohammed Hussein, Rasha Mohammed AbdElmageed

**Affiliations:** 1 Department of Family Medicine, Qatar University Health Center, Duha, QAT; 2 Department of Family Medicine, Primary Health Care Center (PHCC) Qatar, Doha, QAT

**Keywords:** hypothyroidism and type 2 diabetes mellitus, insulin resistance, type 2 diabetes mellitus and thyroid dysfunction, thyroid diseases, type 2 diabetes mellitus

## Abstract

Diabetes and thyroid diseases are caused by endocrine dysfunction and both have been demonstrated to mutually impact each other. Variation in thyroid hormone levels, even within the normal range, can trigger the onset of type 2 diabetes mellitus (T2DM), particularly in people with prediabetes. However, the available evidence is contradictory.

The purpose of this review is to understand the pathological relationship between thyroid-related disorders and T2DM.

T2DM in thyroid dysfunction is thought to be caused by altered gene expression of a group of genes, as well as physiological abnormalities that result in decreased glucose uptake increased, splanchnic glucose absorption, disposal in muscles, increased hepatic glucose output. Additionally, both hyperthyroidism and hypothyroidism can cause insulin resistance. Insulin resistance can develop in subclinical hypothyroidism as a result of a reduced rate of insulin-stimulated glucose transfer caused by a translocation of the glucose transporter type 2 (GLUT 2) gene. On the other hand, novel missense variations in (Thr92Ala) can cause insulin resistance. Furthermore insulin resistance and hyperinsulinemia resulting from diabetes can cause culminate in goitrous transformation of the thyroid gland.

Thyroid-related diseases and T2DM are closely linked. Type 2 diabetes can be exacerbated by thyroid disorders, and diabetes can worsen thyroid dysfunction. Insulin resistance has been found to play a crucial role in both T2DM and thyroid dysfunction. Therefore, failure to recognize inadequate thyroid hormone levels in diabetes and insulin resistance in both conditions can lead to poor management of patients.

## Introduction and background

Thyroid dysfunction and diabetes mellitus are the most frequently occurring endocrinopathies with a large impact on cardiovascular health. Diabetes is a global pandemic. Globally, the prevalence of diabetes has increased as a result of the rise in obesity and lifestyle changes. In 2017, the global prevalence of diabetes mellitus was 425 million. Currently, the worldwide prevalence of diabetes is rising and is expected to reach 366 million by 2030, impacting 44% of all age groups [1]. On the other hand, in the United States and Europe, the prevalence of thyroid dysfunction is 6.6% in adults; it is increasing with age and is more common among women compared to males.

Thyroid disorders are also significantly more prevalent in type 2 diabetes mellitus (T2DM) patients, ranging between 9.9% and 48%. Furthermore, studies have also recorded a high prevalence of thyroid disorder in the 13.4% diabetic population, with a higher prevalence (31.4%) among females with type 2 diabetes than males with T2DM (6.9%) [2]. Evidence also suggests a strong underlying relationship exists between thyroid diseases and diabetes mellitus. Researchers have shown that the thyroid hormone plays a role in controlling glucose metabolism and pancreatic function, while diabetes can alter thyroid function. For instance, “TSH to thyrotropin-releasing hormone response” has been found to be reduced in diabetes, causing accompanying decreased T3 levels and hypothyroidism [3]. It has been proposed during diabetes reduced T3 levels can decrease the conversion of T3 from T4 on the basis of research conducted to observe hyperglycemia triggered reversible decline in hepatic thyroxine concentration and deiodinase activity. Other studies have revealed that increased T3 levels even for short period can cause insulin resistance; thereby contributing to T2DM.

Numerous investigations also have shown that an array of intricately associated hormonal, genetic, and biochemical abnormalities mimic this pathophysiological relationship. For example, the “5′ adenosine monophosphate-activated protein kinase” (AMPK) is the main target for altering thyroid hormone feedback and insulin sensitivity regulation linked with energy expenditure and appetite [4]. Additionally, hypothyroidism (for example, Hashimoto’s thyroiditis) and hyperthyroidism (for instance, Graves' disease) have been linked to diabetes mellitus. According to a comprehensive study, thyroid dysfunction occurs at a rate of 11% in diabetic patients. Moreover, autoimmunity has been identified as the primary etiology of diabetes mellitus linked with thyroid disorders. Furthermore, certain genetic variations have also been found associated with thyroid disorders and T2DM, for instance, mutations in GLUT4.

However, the link between T2DM and related thyroid disorders remains highly debatable and human research have shown conflicting results. Therefore, the purpose of this review is to understand the pathological relationship between thyroid-related disorders and T2DM.

## Review

The Link between thyroid disorders and T2DM

Thyroid hormones exert a direct influence on insulin secretion. Hypothyroidism resulted in a decrease in insulin production via beta cells whereas hyperthyroidism led to an increase in beta-cell responsiveness to catecholamine or glucose due to increased beta-cell mass. Additionally, thyrotoxicosis results in an increase in insulin clearance [5]. All of these changes occur as a result of alternations in thyroid hormone which increases the risk of developing T2DM and can lead to diabetic complications or can worsen diabetic symptoms.

Hyperthyroidism and T2DM

Increased glucose production from the liver is a key factor in the development of peripheral insulin resistance, glucose intolerance, and hyperinsulinemia [6]. In thyrotoxicosis, glucose tolerance is triggered by an increase in hepatic glucose output and an increase in glycogenolysis [7]. This process contributes to the progression of subclinical diabetes and exacerbation of hyperglycemia in type 2 diabetes. Studies have also reported that both T2DM and hyperthyroidism share some pathological features. For example, TD2M is characterized by changes in B-cell mass, decreased insulin secretion, and elevation in intestinal glucose absorption, upsurge in glucagon secretion, increase in insulin breakdown, insulin resistance, and increased levels of catecholamines. These factors are also an important part of hyperthyroidism [8]. Among the aforementioned factors, insulin resistance has been identified as the most significant link between thyroid malfunction and T2DM. Hepatic insulin resistance is promulgated by excessive production of glucose rather than fasting hyperinsulinemia. Additionally, increased hepatic glucose output has been found to be a critical regulator of elevated fasting plasma glucose (FPG) concentrations in T2DM patients [9]. During insulin resistance, muscle glucose is increased although uptake efficiency is decreased. Reduced glucose uptake into muscles and increased hepatic glucose output result in a deterioration of glucose metabolism. It is worth noting that insulin resistance can occur in both hyperthyroidism and hypothyroidism. According to recent discoveries, insulin resistance also impairs lipid metabolism [10]. Thus, insulin resistance appears to be a possible connection between thyroid dysfunction and T2DM.

Similarly, another study showed that beta-cell dysfunction and insulin resistance both are negatively connected to TSH, which may be explained by thyroid hormones' insulin-antagonistic properties combined with a rise in TSH. A higher blood T3 and T4 levels often result in decreased TSH levels via a negative feedback process. Thyroid hormone levels drop as TSH levels decrease and insulin-antagonistic effects are diminished and when TSH level decreases, thyroid levels increase and insulin-antagonistic effects are elevated. However, the mechanism by which hyperthyroidism leads to insulin resistance is not known but it is the most commonly occurring phenomenon observed in diabetic patients with hyperthyroidism.

Genetic Variations, Hyperthyroidism, and T2DM

Following genes named mitochondrial uncoupling protein, GLUT4, GLUT1, “PPAR gamma coactivator-1 alpha (PGC-1 alpha)”, phosphoglycerate kinase (PGK), [11] regulate the connection between thyroid hormone with skeletal muscles. Among the several discovered genes, UCP-3 and GLUT-4 have been extensively researched. In skeletal muscles, T3 mediates GLUT-4 and it has been revealed to increase basal and insulin-induced glucose transport [12]. “Mitochondrial uncoupling protein 3” (UCP 3) is a newly found gene that has been linked to reducing fatty acid oxidation and glucose metabolism [13]. Additionally, research has reported that this gene plays a significant role in the downregulation of “5′ adenosine monophosphate-activated protein kinase and Akt/PKB signaling” [14]. Furthermore, the potential role of T2 has been investigated, and it has been established that it is connected with sarcolemma GLUT-4. Similarly, glycolytic enzymes and phosphofructokinase have been linked to GLUT 4 activity mediated by T2 [14]. Numerous genes have also been identified as being involved in peripheral glucose metabolism.

For example, T3 activates an array of genes involved in glucose metabolism that attaches to the thyroid hormone receptors. These receptors originate from TRβ1, TRβ2, TRβ3, and TRα1, respectively. These are the four primary isoforms of T3 binding [15]. TR α1 is believed to modulate thyroid hormones' metabolic actions. TRβ2 and TRβ1 are associated with the maintenance of the “hypothalamic-pituitary-thyroid axis” and the maintenance of a normal thyroid function [16].

Similarly, 3,5,3-triiodothyronine originate from T4. It can be triggered by the type 2 or type 1 iodothyronine deiodinases type via removal of the iodine atom from the phenolic ring (D2). On the other hand, type 3 deiodinase (D3) inactivates thyroid hormone by removing an iodine atom from the tyrosyl ring. Deiodinase are involved in the regulation of T3 bioavailability and therefore the insulin response. Thyroid hormones influence the expression of deiodinases in various tissues. They regulate T3 bioavailability and hence insulin responsiveness. T3 elevations are linked to a new missense variation (Thr92Ala). This is linked to insulin resistance. Additionally, it is related to an increase in insulin-induced glucose clearance and glucose turnover in skeletal muscle and adipose tissue. In a meta-analysis, it was determined that “intracellular tri-iodothyronine” (T3) is associated with insulin sensitivity abnormalities [16]. Research revealed that expression of GLUT 2 was enhanced in hyperthyroidism compared to the euthyroid phase [17]. Moreover, disturbances in lipid metabolism further establish a relationship between TH and insulin resistance in [17]. Furthermore, thyrotoxicosis results in an increase in lipid peroxidation, while hypothyroidism results in a decrease in glucose oxidation. Reduced LDL cholesterol and triglyceride levels result from LDL clearance. TH stimulates catecholamine activity, resulting in lipolysis of adipocytes and an increase in circulating FA. Increased FA supply counteracts the TH-mediated enhancement of the hepatic long-chain FA oxidative pathway which is involved in glucogenesis. All these genes associated with thyroid hormones play an important part in the pathogenesis of type 2 diabetes.

Hypothyroidism and T2DM

Hypothyroidism is characterized by decreased glucose absorption from the GI tract, extended peripheral glucose buildup, gluconeogenesis, decreased hepatic glucose production, and decreased glucose disposal [18]. Hypothyroidism can affect glucose metabolism in type 2 diabetes in different ways. For example, subclinical hypothyroidism can result in insulin resistance due to a decreased rate of insulin-stimulated glucose transfer induced by a translocation of the GLUT 2 gene. Additionally, according to a study, in hypothyroidism due to decreased insulin clearance by the kidneys, the physiological need for insulin was decreased. Moreover, anorectic circumstances may also contribute to lower insulin production in hypothyroidism.

Furthermore, insulin resistance has been linked to hypothyroidism in a number of preclinical and in vitro investigations [19], where it was discovered that peripheral muscles become less sensitive to insulin under hypothyroid conditions. A plausible role for such disease has been suggested by dysregulated leptin metabolism [20]. Additionally, numerous authors have established a direct link between insulin resistance and hypothyroidism [21]. However, some researchers have observed inconsistent findings, highlighting the need for more research in this area.

Thyroid diseases and T2DM

The link between T2DM and thyroid cancer incidence is debatable. Large prospective cohort research discovered an increased risk of differentiated thyroid cancer among type 2 diabetic women [22]. Another large prospective study and a pooled analysis of numerous prospective trials [23] revealed no evidence of a significant relationship between thyroid cancer and diabetes. Additionally, a prior review of the literature revealed that any link between thyroid cancer and T2DM was most likely weak [24]. However, Korean research found that patients with early T2DM had a low incidence of thyroid cancer, with the effect continuing up to six years after the T2DM was detected [25]. Moreover, according to retrospective research published in December 2018, Chinese women with T2DM had a considerably increased risk of thyroid cancer [26].

Furthermore, evidence indicates that subclinical hypothyroidism or hyperthyroidism raises blood pressure and cholesterol levels, impairs insulin secretion, and compromises both micro-and macrovascular function, increasing the risk of peripheral neuropathy, peripheral artery disease, and diabetic nephropathy. On the other hand, another study proposed that SCH can protect against cardiovascular death in T2DM. Additionally, a previous review explored the relationship between diabetic complications and subclinical hypothyroidism. This meta-analysis discovered that T2DM patients having subclinical hypothyroidism were at increased risk of developing diabetic complications including peripheral neuropathy, nephropathy, and retinopathy. From the above findings, it is safe to assume that thyroid diseases can increase the risk of diabetic complications or can worsen diabetic symptoms. However, future research on the relationship between thyroid cancer and diabetes mellitus is highly recommended.

Effect of type 2 diabetes on thyroid diseases

In type 2 diabetes, older age, obesity, and female sex, hospitalization, and thyroid peroxidase antibody Ab positive all enhance the risk of developing hypothyroidism. Diabetes impairs thyroid function by changing thyroid-stimulating hormone (TSH) levels and by disturbing the conversion of thyroxine (T4) to triiodothyronine (T3) in peripheral tissues [27]. In euthyroid diabetic patients, the nocturnal TSH peak can be absent or diminished and the TSH response to thyrotropin-releasing hormone (TRH) can be compromised. However, long-term hyperglycemia can have a cumulative effect on thyroid dysfunction. Therefore, while interpreting thyroid function tests, it is critical to keep in mind that, similar to other acute systemic diseases, diabetic ketoacidosis can result in a drop in T3 and T4 levels while TSH levels can stay normal. Moreover, hyperinsulinemia and Insulin resistance promote thyroid tissue proliferation, increase the prevalence of nodular thyroid disease, and result in a goiter [28]. Additionally, diabetic individuals with goiter orbitopathy are at more risk of developing dysthyroid optic neuropathy than nondiabetics. Numerous researches also have shown that the association between diabetes and thyroid function can be bidirectional. For example, early type 2 diabetes or prediabetes can increase thyroid tissue hyperplasia, resulting in enlargement of the thyroid gland and development of nodules. On the other hand, thyroid dysfunction affects glucose metabolism in diabetes. Furthermore, it is well established that the prevalence of subclinical hypothyroidism increases with age. Females and males have distinct thyroid dysfunction predispositions and obesity has been demonstrated to be strongly associated with hypothyroidism [29]. A review of 36 studies concluded that T2DM females over the age of 60 have a greater prevalence of subclinical hypothyroidism. Furthermore, in India, a cross-sectional observational study observed 1,508 T2DM patients and they discovered a significantly elevated risk of hypothyroidism in older type 2 diabetic patients (more than 65 years) with an OR of 4.2, and a clear difference between females and males (OR 4.82 vs 2.60), as well as between obese and normal patients (OR 2.56 vs. 3.11) [30]. This implies that BMI status, gender, age, and sex hormones can also have a role in thyroid dysfunction and T2DM.

## Conclusions

 

There is much evidence that thyroid disease and T2DM are closely related. T2DM is characterized by changes in beta-cell mass, decreased insulin secretion, and elevation in intestinal glucose absorption, upsurge in glucagon secretion, increase in insulin breakdown, insulin resistance, and increased levels of catecholamines. These factors are also an important part of hyperthyroidism.

Additionally, the existing evidence demonstrates that insulin resistance plays a critical role in the connection between thyroid dysfunction and T2DM. Both thyroid dysfunction and T2DM have a bidirectional relationship. Thyroid disorders such as thyrotoxicosis and hypothyroidism can cause insulin resistance. Insulin resistance can develop in subclinical hypothyroidism as a result of a reduced rate of insulin-stimulated glucose transfer caused by a translocation of the glucose transporter type 2 gene (GLUT 2). On the other hand, higher levels of T3 activate a number of genes involved in glucose metabolism and insulin resistance. Additionally, insulin resistance and hyperinsulinemia enhance thyroid tissue development, which can cause nodular thyroid disease and a goiter. Furthermore, the literature suggests that subclinical hypothyroidism or hyperthyroidism raises blood pressure and cholesterol levels, impairs insulin secretion, and compromises both micro- and macrovascular function, increasing the risk of peripheral neuropathy, peripheral artery disease, and diabetic nephropathy. All these findings suggest that a strong relationship exists between thyroid diseases and TD2M and by early screening or by recognizing the risk factors, the risk of these two conditions and their medical complications can be minimized.
